# COVID Transitions: Characteristics of older adults who engage or do not engage in virtual exercise

**DOI:** 10.1093/geroni/igaa057.3434

**Published:** 2020-12-16

**Authors:** Catalin Mateas, Janet Bettger, Stephen Jennings, Kenneth Manning, Katherine Hall, Megan Pearson, Miriam Morey

**Affiliations:** 1 Durham VA Health Care System, Durham, North Carolina, United States; 2 Duke University School of Medicine, Durham, North Carolina, United States; 3 Durham VA Geriatric Research, Education, and Clinical Center (GRECC), Veterans Affairs Health Care System, Durham, North Carolina, United States; 4 Duke University, Veterans Health Administration, Durham, North Carolina, United States; 5 Veteran Affairs Health Care System, Durham, North Carolina, United States; 6 Veterans Health Administration, Durham, North Carolina, United States

## Abstract

Background. Exercise is a crucial component of maintaining good health in older individuals. The COVID-19 stay-at-home orders forced Veterans actively engaged in facility-based exercise to stop attending in-person group exercise programs like Gerofit. Objective. To compare the characteristics of Veterans who enrolled (E) or declined enrollment (DE) in the transition from a facility-based exercise program, Gerofit, to a virtual Gerofit-to-Home (GTH) program. Methods. Gerofit is a supervised exercise “VA Best Practice” program for older Veterans implemented at 17 VA medical centers around the country. At the time of COVID-19 mandated closures, 1149 Veterans were actively engaged in facility-based programs and invited to attend GTH classes. Comparisons between those enrolling and those declining enrollment were performed by t-tests. Results. Three hundred and eight of 1149 (27%) Veterans made the transition to telehealth delivered classes, with several sites having enrolled participants aged in their mid-nineties. Age was not associated with GTH adoption rates (74.0 vs. 74.7, p=not significant for E vs. NE). Body mass index (31.3 vs. 30.5 kg/m2, p<0.05), gait speed (1.19 vs. 1.12 m/s, p<0.001), arm curls (20.8 vs. 19.5, p<0.001), and chair stands (14.7 vs. 13.2, p<0.05) were higher in individuals actively participating in GTH compared to those that never enrolled. Conclusions. Some older adults can adopt a virtual approach to group-based exercise, demonstrating its feasibility. Further research is needed to improve GTH implementation for lower functioning individuals. Virtual group-based exercise could reduce negative health effects associated with isolation due to lack of in-person exercise.

